# Macromolecular Design Principles Governing Electrospinning of Polymer Nanofibers

**DOI:** 10.3390/polym18080929

**Published:** 2026-04-10

**Authors:** Lan Yi, Christian Dreyer

**Affiliations:** 1 Department Fiber Composite Material Technologies, Faculty of Engineering and Natural Sciences, Technical University of Applied Sciences Wildau, Hochschulring 1, 15745 Wildau, Germany; 2 Research Division Polymeric Materials and Composites PYCO, Fraunhofer-Institute for Applied Polymer Research, Schmiedestr. 5, 15745 Wildau, Germany

**Keywords:** electrospinning, macromolecular design, chain entanglement, molecular weight distribution, polymer dynamics, nanofiber scaling

## Abstract

Electrospinning is a versatile technique for producing polymer nanofibers with high ratios of surface area to volume and tunable porosity. Conventional approach to the optimization of processing parameters such as voltage and flow rate frequently encounters limitations in reproducibility and scalability. This review proposes a comprehensive framework that integrates macromolecular design principles with established electrohydrodynamic theories. We analyze how intrinsic molecular traits, specifically chain entanglement density, molecular weight distribution (MWD), topological architecture, and polymer–solvent thermodynamic interactions, define the boundaries of jet stability and solidification. Key findings highlight that while molecular weight establishes a baseline for spinnability, the MWD dictates the dynamic response under extreme deformation. Notably, high-molecular-weight fractions act as elastic load-bearers that suppress capillary breakup. Furthermore, we discuss here how molecular architecture and solvent-mediated segmental mobility determine whether molecular orientation is kinetically trapped or relaxed during the nanosecond timescales of jet flight. By establishing a hierarchical design logic prioritizing molecular and formulation variables over processing parameters, this framework provides a robust strategy to overcome challenges in scalability and reproducibility, positioning electrospinning as a sensitive probe for macromolecular dynamics under extreme elongation.

## 1. Introduction

Electrospinning has become one of the most versatile routes for producing polymer micro- and nanofibers with a high surface-area-to-volume ratio, tunable porosity, and diverse structural functionality. Owing to its relative experimental simplicity and compatibility with a wide range of polymer systems, electrospinning has been widely explored for membrane separation, energy related materials, biomedical scaffolds, and composite reinforcement [1,2,3]. Despite this broad utility, electrospinning is still frequently optimized through empirical adjustment of processing variables such as applied voltage, flow rate, and collector configuration. Although these parameters undoubtedly influence jet behavior and fiber morphology, their apparent effects often vary strongly across polymer–solvent systems and experimental setups, which limits reproducibility, transferability, and rational scale-up [3,4,5,6,7,8].

A growing body of evidence suggests that these difficulties cannot be understood solely in terms of processing parameters. Rather, they reflect the fact that electrospinning is fundamentally constrained by macromolecular properties that govern how polymer solutions respond to extreme elongational deformation [9,10,11,12,13,14,15,16,17,18]. In particular, chain entanglement density [19,20,21,22], molecular weight distribution [23,24,25], chain architecture [26,27], intermolecular interactions [28,29,30,31,32], and polymer–solvent thermodynamic compatibility collectively determine whether a charged jet can sustain stretching, suppress capillary breakup, and solidify into continuous fibers [33,34,35]. From this perspective, electrospinning is not only a fabrication technique but also a sensitive probe of macromolecular dynamics under highly nonequilibrium conditions. Previous reviews have provided valuable insight into electrospinning by emphasizing process optimization, equipment design, fiber applications, or morphology control. However, the role of macromolecular design is often treated in a fragmented way, with polymer chemistry, solution behavior, and jet stability discussed separately rather than as parts of a unified framework [11,36,37,38,39,40,41,42,43,44,45]. As a result, the literature still lacks a sufficiently integrated review that explains how intrinsic molecular descriptors define the accessible electrospinning window and the morphology space that can be reliably obtained.

This review therefore adopts a macromolecular design-centered perspective on electrospinning. Specifically, we focus on how molecular weight, molecular weight distribution, chain architecture, intermolecular interactions, relaxation dynamics, and polymer–solvent interactions govern spinnability through their coupled effects on entanglement development, stress transmission, jet stability, and solidification. The purpose of this review is not to comprehensively summarize all operational or device level aspects of electrospinning, nor to survey all application fields in equal depth. Instead, we concentrate on those material and formulation variables that directly explain why chemically or structurally different polymers exhibit distinct spinning behavior and fiber morphologies even under nominally similar processing conditions. The manuscript is organized accordingly. Section 2 examines the molecular level foundations of electrospinning, with emphasis on chain entanglement, molecular weight distribution, architecture, relaxation behavior, and polymer–solvent interactions. Section 3 translates these concepts into a hierarchical design framework in which molecular and formulation variables are considered prior to processing optimization. Section 4 discusses the broader implications of this perspective for reproducibility, scalability, and future polymer design strategies. Through this structure, the review aims to move beyond descriptive parameter tuning toward a more mechanistic and transferable understanding of electrospinning.

## 2. Macromolecular Engineering: Chemistry and Chain Dynamics

The transition from a polymer solution to a solidified nanofiber involves elongation flow occurring on a very short timescale. Understanding polymer molecular behavior during this non-equilibrium transition is essential for explaining electrospinning-induced structure formation.

### 2.1. Chain Entanglement

**Figure 1:**
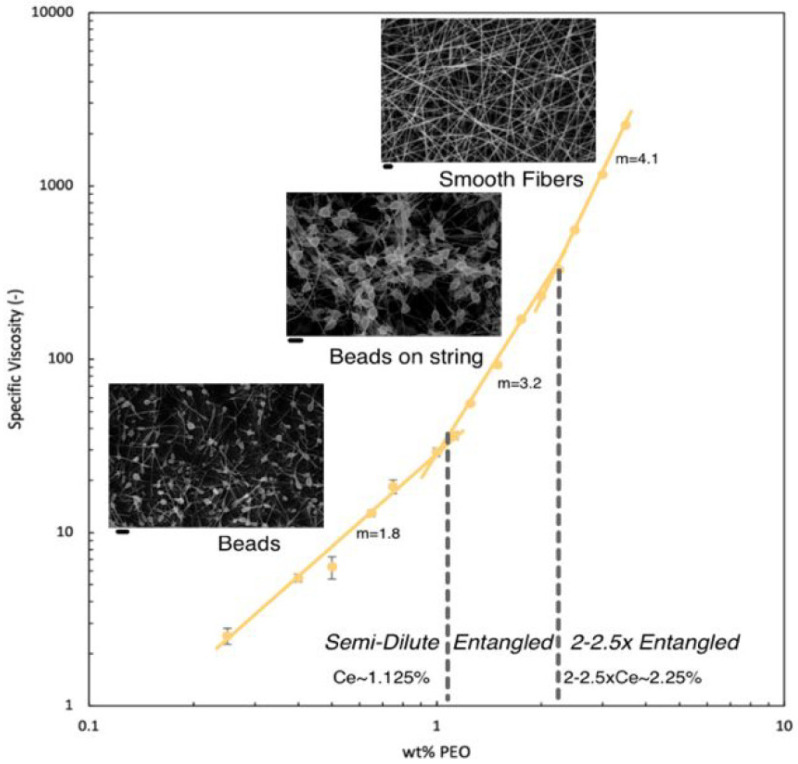
Morphological transition during electrospinning governed by chain entanglement. Representative fiber morphologies and viscosity-scaling regimes illustrating the progression from droplets or bead-containing structures to uniform continuous fibers as polymer concentration, entanglement density, and viscoelastic stress increase. The changes in the fitted slope m of the log–log plot of specific viscosity versus polymer concentration indicate transitions between concentration regimes associated with electrospraying, beads-on-string formation, and stable fiber formation. Adapted from [75] under CC BY-NC-ND 4.0.

Chain entanglement provides the transient network elasticity required to suppress capillary breakup under elongation flow, which is an important molecular design parameter [46,47,48,49,50,51,52]. The macromolecular network must also be able to sustain significant elastic stresses during the fast jet stretching. If the intermolecular connectivity is not sufficient, the jet becomes unstable and fails to Rayleigh instabilities leading to electrospraying or beads-on-string morphologies [46,47,49,53,54]. Early electrospinning studies relied primarily on weight concentration as an indicator of spinnability [55,56,57]. However, the concentration alone cannot account for the thermodynamics of chain dimension and consequently cannot be generalized across diverse polymer–solvent systems. Polymers with identical concentration but differing molecular weight ($M_{w}$) [58,59,60,61], molecular architecture (e.g., linear versus branched) [9,60,62,63,64,65] or solvent quality (usually defined by Flory–Huggins interaction parameter) [66,67,68] often exhibit different electrospinning behavior. The studies on polycaprolactone (PCL) [69,70,71,72] illustrate that solutions prepared even at identical concentration (10 wt/v% ) showed obviously different electrospinning behavior based on the solvent choice. Namely, uniform fibers were obtained in chloroform while bead formation dominated in DMF/acetone mixtures. It was attributed to differences in solvent, which modulated the onset of chain overlap and effective entanglement. These observations draw attention to entanglement-based concept which is the critical entanglement concentration ($C_{e}$) concept, which marks the transition from a semi-dilute unentangled to a semi-dilute entangled solution [73]. Stable fiber formation is typically observed only when the solution concentration reaches a normalized threshold based on the framework [74]. This concentration-dependent transition from electrospinning to continuous fiber formation is illustrated in Figure 1.

The dimensionless overlap parameter [η]C, defined as the product of intrinsic viscosity [η] and polymer concentration *C*, has been widely used as a scaling parameter to compare electrospinning behavior across polymer systems with different molecular weights. This parameter reflects the degree of molecular overlap in solution and provides a unified framework for describing the transition from electrospraying or bead formation to continuous fiber formation [22]. Experimental studies on model linear polymers like poly(ethylene oxide) (PEO) [74,76] and polyacrylonitrile (PAN) [77] have shown that the morphology transitions from droplets to beads and ultimately to uniform fibers correlate with increasing values of [η]C. Although no universal critical value exists, stable fiber formation has frequently been reported when [η]C reaches values in the order of several times unity, consistent with emergence of a sufficiently interconnected entanglement network capable of resisting capillary breakup. Branched or star-shaped polymers often require higher numbers to achieve the same jet stability as linear polymers because their compact hydrodynamic volume reduces the probability of effective inter-chain entanglement [78,79]. The optimum entanglement density is critically affected by the macromolecular relaxation time $\tau_{Z}$ [80]. The entanglements act as virtual crosslinks if the whipping jet process time is shorter than the polymer’s relaxation time (characterized by Deborah number (De)) [81,82]. These kinetic entanglements lock the structure in a non-equilibrium state upon solidification. Research [83,84] now explores the polymer-free electrospinning of supramolecular systems, where traditional covalent entanglements are replaced by high-density hydrogen bond or π-π stacking in order to achieve fiber stability through pseudo-entanglements that mimic the rheological behavior or high molecular weight polymers [85,86,87].

### 2.2. Molecular Weight Distribution and Polydispersity Effect

While the weight-average molecular weight ($M_{w}$) establishes the baseline requirement for chain entanglement, the molecular weight distribution (MWD) as determined by polydispersity index (PDI) influences the dynamic response of polymer solutions under the elongational strain rates, which are characteristics of the electrospinning jet [36,74,85]. The polymers with narrow MWD were favored for their predictable rheological behavior [85], but new publications suggest that engineered polydispersity acts as a stabilizing tool to expand the spinnable window by providing a broader spectrum of relaxation time [74,85,88].

#### 2.2.1. Role of High-Molecular-Weight Fractions in Jet Stabilization

**Figure 2:**
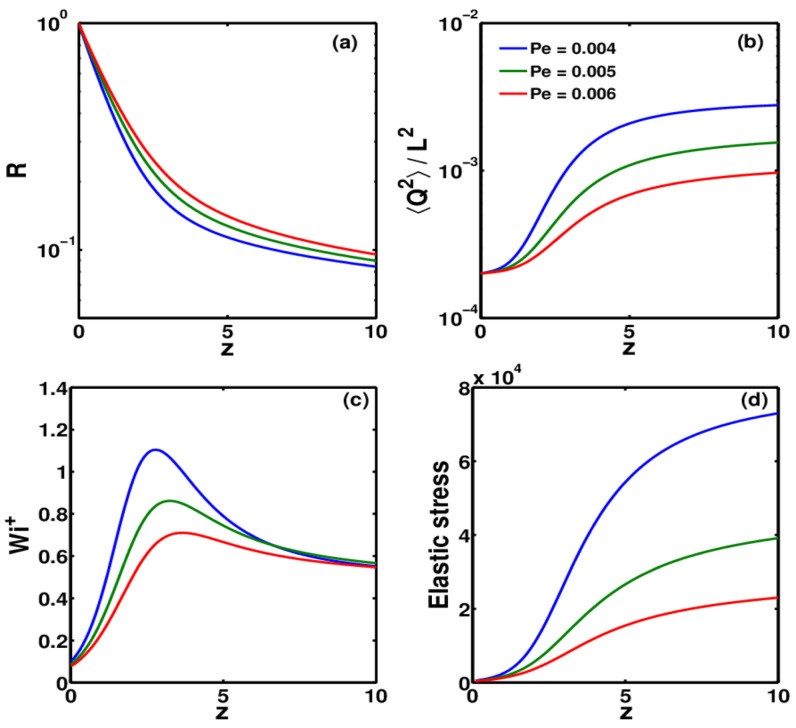
Development of viscoelastic stresses and polymer chain stretching along the electrospinning jet as a function of relaxation time. Increasing relaxation time enhances elastic stress growth and promotes coil–stretch transition of polymer chains, enabling suppression of capillary breakup and stabilization of continuous fiber formation. Effect of the electric Peclet number (Pe) on the evolution of electrospinning jet dynamics along the axial distance (z) from the nozzle to the onset of the whipping region. (**a**) Variation of the jet radius (R) as a function of axial position z, illustrating the thinning behavior of the electrified jet for polymer solutions with different Pe numbers. Higher Pe values correspond to faster jet thinning in the Taylor cone region. (**b**) Evolution of the normalized molecular extension ⟨Q^2^⟩/L^2^, representing the degree of polymer chain stretching during jet elongation, where a value approaching unity indicates nearly fully stretched polymer chains. (**c**) Variation of the local Weissenberg number (Wi^+^), which characterizes the ratio of polymer relaxation time to the local deformation rate and reflects the strength of extensional flow experienced by the jet. (**d**) Development of elastic stress (ES) along the jet axis, demonstrating how stronger stretching and higher Wi^+^ promote the growth of elastic stresses that can stabilize the jet and suppress capillary breakup. Adapted from [82] under CC BY- 4.0 license.

High-molecular-weight fractions within a polydisperse polymer play a disproportionate role in determining the extensional rheological response of electrospinning solutions [74,88,89]. Shorter chains in such systems contribute to improved processability by reducing the overall solution viscosity and facilitating flow through the spinneret but ultra-high-molecular-weight fractions act as elastic load-bearing components [85,90,91]. These long chains have relaxation times that can exceed the characteristic deformation timescale of the electrospinning jet. They experience a coil to stretch transition as the jet undergoes rapid thinning, storing elastic energy and generating the stabilizing viscoelastic stress required to reduce Rayleigh instabilities [90,91]. The evolution of elastic stress and polymer chain stretching during jet extension is illustrated in Figure 2 [82]. This mechanism affects polyelectrolyte and biopolymers like chitosan [92,93], where adding trace amounts of high molecular weight PEO allows for stabilization of spinning at total concentrations significantly lower than those required for monodisperse samples [74,93,94]. This behavior reflects from a macromolecular perspective the partial decoupling of bulk viscosity from extensional elasticity. Whereas viscosity is influenced by the full molecular weight distribution, extensional stress development is often dominated by the longest relaxation modes associated with the high-molecular-weight tail. Consequently, manipulation of molecular weight distribution provides a strategy to enhance jet stability without proportionally increasing shear viscosity or processing resistance [90,91].

#### 2.2.2. Influence of Polydispersity on Fiber Morphology and Uniformity

The MWD dictates not only jet stability but the distribution of fiber diameters. A broad PDI can lead to bimodal fiber populations, a tuned MWD can reduce the formation of beads and improve morphological uniformity [74,94,95]. The self-stabilizing behavior arises from the strain hardening response inherent to polydisperse solutions [91]. The localized increase in elongational stress preferentially stretches the longest chains as the local necking starts along the jet. This leads to a localized increase in extensional viscosity confirmed by extensional rheometric studies [91]. A broader MWD has been shown to result in a more uniform self-healing jet for polymers like PAN [96,97]. The strain-hardening of the high $M_{w}$ tails stops the necking before it leads to jet breakup or bead formation [74,95]. This broad relaxation spectrum ensures that the jet can respond to a wider range of temporal instabilities during the high frequency whipping stage [85,91].

#### 2.2.3. Implications for Circular Polymer Design and Industrial Upcycling

The role of MWD is critical in the context of circular economy. Recycled polymers like poly(ethylene terephthalate) (PET) [98,99] and polystyrene (PS) [100,101] suffer from chain scission during their initial life cycle resulting in a loss of the high $M_{w}$ fractions required for the entanglement [98,100]. Numerous studies have demonstrated that the spinnability of degraded polymer streams can be partially recovered through molecular weight restoration strategies. This includes the blending with high-molecular-weight fractions or reconstructing the entanglement network, thereby re-establishing the viscoelastic response required for stable jet formation [36,86,94]. Experimental studies on recycled PET and related polymers have shown that supplementation of the high-molecular-weight tail can recover extensional elasticity and re-establish stable jet formation. This highlights how MWD engineering bridges the gap between the irregular molecular spectra of waste plastics and the precision required for high-performance nanofiber fabrication. It also strengthens the idea that electrospinning performance is a function of the entire molecular weight spectrum rather than a single average value [36,74,85,102]. The key macromolecular parameters discussed throughout this section and their respective roles in electrospinning behavior are summarized in Table 1.

### 2.3. Molecular Architecture

The molecular architecture of polymers introduces topological constraints that influence chain connectivity, relaxation dynamics, and stress transmission during the electrospinning process, in addition to effect of molecular weight and its distribution. The molecular architecture ranging from linear and branch to star-shaped or block copolymer structures dominate the hydrodynamic volume, and the probability of entanglement overlap, even at comparable weight-average molecular weights [20,85,107,108,109].

#### 2.3.1. Architecture Control of Entanglement Efficiency

Linear polymers generally exhibit the most efficient entanglement per unit weight as their extended conformations maximize the radius of gyration relative to their molecular weight. In contrast, branched and star-shaped polymers are characterized by a Zimm–Stockmayer branching index $g=\frac{R_{g,\mathrm{branched}}^{2}}{R_{g,\mathrm{linear}}^{2}}$, where g < 1 indicates a more compact conformation [85,110,111,112]. The star-shaped polymers exhibit higher critical entanglement concentrations because they have a smaller hydrodynamic radius at an equivalent $M_{w}$ [113,114,115]. Experimental reports on star-branched PCL indicate that stable fiber formation typically requires higher concentrations than for linear analogues of similar molecular weight, reflecting the reduced coil dimensions and diminished intermolecular overlap associated with branched topologies [116,117,118]. But hyperbranched polymers [119] and dendrimers [120,121] often fail to spin into continuous fibers even at ultra-high concentrations due to their globular and non-entangling nature, unless they are blended with linear high-$M_{w}$ samples to provide the necessary elastic connectivity [119].

#### 2.3.2. Block Copolymers and Nonequilibrium Phase Separation

Block copolymers [102,103] introduce a chemical level that drives microphase separation during nanosecond flight time of the jet. Previous studies have shown that electrospinning of block copolymers can produce internal nanodomains within the fibers [1]. The high-strain-rate elongation competes with the thermodynamic drive for the block to segregate according to Flory–Huggins interaction parameter [59,122]. Electrospinning occurs under strongly nonequilibrium conditions characterized by rapid solvent evaporation and short jet residence times, which can preserve transient structural organization and effectively freeze morphologies formed during elongational flow [36,37,123]. The electrospinning has been shown to produce nanofibers with microphase-separated domains in the block copolymer systems. These can become oriented along the fiber axis under extensional deformation [1,124,125]. Such alignment has attracted interest for applications requiring anisotropic transport properties, including ion-conducting membranes. Selective solvent electrospinning can further induce internal compositional asymmetry, producing Janus or multicore fiber architectures in which morphology is governed by block compatibility and solvent selectivity [126,127,128].

#### 2.3.3. Topological Constraints and Relaxation Pathways

Molecular architecture features also influence electrospinning behavior by modifying polymer relaxation pathways under high-strain-rate deformation. Polymers with complex topologies exhibit multiple relaxation modes like arm retraction, branch-point motion or block-specific dynamics [85,88,129,130]. This additional relaxation mechanism can either stabilize or destabilize the jet depending on their characteristic timescales relative to the electrospinning process [131,132]. When architecture relaxation times are long compared to characteristic deformation time of jet, topological constraints act as transient elastic junctions to enhance resistance to capillary instabilities. The arm retraction time in star-shaped polymers can be significantly longer than the process time of the whipping jet and it will cause the arm act as transient elastic junctions [130,132,133]. It effectively increases the elongational viscosity and prevents jet breakup. But architectures that relax rapidly may dissipate stress before solidification and reduce jet stability despite adequate entanglement density. The dense grafting of side chains limits backbone entanglement in highly branched polymers [131,134,135] while increasing chain rigidity. Studies showed that these architectures can produce ultra-soft and super-elastic nanofiber mats because the architecture prevents the formation of dense and rigid crystalline domains maintaining a permanently amorphous and rubbery state.

#### 2.3.4. Architecture Design for Electrospinning Control

In summary, the transition from linear macromolecules to complex topologies transforms polymer architecture into a primary lever for controlling fiber formation. Architecture unlike molecular weight allows for the decoupling of spinnability from bulk solutions which primarily affects the magnitude of entanglement [136]. This provides for a multidimensional framework for the design of electrospinnable system, where synthetic decisions at the molecular level can be determined in the following ways. (1) By manipulating the branching index and chain length and tune the elongation viscosity and strain-hardening behavior of a solution. This allows for the optimization of high-throughput processes such as needle-less electrospinning where star-shaped or branched architectures can maintain jet stability at lower pumping pressure or higher concentrations than their linear counterparts [137,138]. (2) The use of block copolymer architectures moves the field beyond simple passive fibers [1,103]. As discussed in Section 2.3.3, the architectural connectivity of immiscible blocks allows for kinetic trapping of functional nanodomains, it makes it possible to design fibers with built-in features like internal nano-channels transportation or Janus-like surface asymmetries governed by chemical composition established during synthesis.

### 2.4. Chain Relaxation and Nonequilibrium Dynamics

In electrospinning, polymer chains are exposed to extreme elongational deformation over short timescales [123,138]. Under these conditions, the ability of a material to form continuous fibers is not dictated solely by processing parameters but by the intrinsic relaxation dynamics encoded in its molecular structure. Fiber formation therefore reflects the balance between externally imposed stretching and chemically defined molecular mobility [36,139]. Under these conditions, the ability of polymer chains to relax is not determined only by macroscopic rheological parameters but rooted in chemically defined features like backbone flexibility, segmental mobility, and intermolecular interactions.

#### 2.4.1. Chemical Constraints on Molecular Flexibility

The primary factor determining relaxation behavior is the internal chemical structure of the backbone. Flexible polymers with low internal rotation barriers possess high segmental mobility [108]. This enables the chains to adjust their shape quickly during the electrospinning process. Polymers containing bulky side groups or aromatic moieties generally exhibit reduced segmental mobility due to increased rotational barriers and internal friction along the backbone. Polystyrene (PS), for example, possesses a relatively stiff backbone associated with its phenyl substituents. Poly(methyl methacrylate) (PMMA), while not classified as a rigid-rod polymer, still exhibits slower segmental dynamics compared with highly flexible chains such as poly(ethylene oxide), owing to steric hindrance from its pendant ester groups [85]. Due to the stiffness of the molecules, chains take much longer to move. In polyacrylonitrile (PAN), strong dipolar interactions between nitrile groups promote intermolecular cohesion and help stabilize chain alignment during high-strain-rate stretching. When deformation outpaces relaxation, these interactions facilitate the retention of molecular orientation prior to solidification [50,82,85,123]. The preserved alignment in electrospun PAN fibers is particularly significant because it defines the precursor structure for subsequent thermal stabilization and carbonization, processes that translate molecular orientation into macroscopic mechanical performance [26,140].

#### 2.4.2. Intermolecular Interactions

Segmental mobility is influenced by intermolecular interactions such as hydrogen bonding, dipole–dipole interactions, and ionic associations. Strong intermolecular attractions effectively increase the lifetime of entanglements by limiting local chain motion, thereby extending relaxation times under deformation. The studies have shown that these chemical attractions lock the entanglements in place while the jet is flying [85,139,141,142]. This prevents the polymer from forgetting its stretched shape before it hits the collector. The specific amino-acid sequence in recombinant spider silk generates a hierarchy of intermolecular interactions that enables elastic energy storage and delays jet breakup under extreme stretching. Consequently, polymers with comparable molecular weight and architecture may still exhibit markedly different electrospinning behavior depending on their interaction chemistry and the extent of transient physical cross-linking in solution [50,143]. As a result, the polymers with comparable molecular weight and architecture may exhibit markedly different electrospinning behavior depending on their interaction chemistry and the degree of transient physical cross-linking present in the solution [144].

#### 2.4.3. Nonequilibrium Conditions

**Figure 3:**
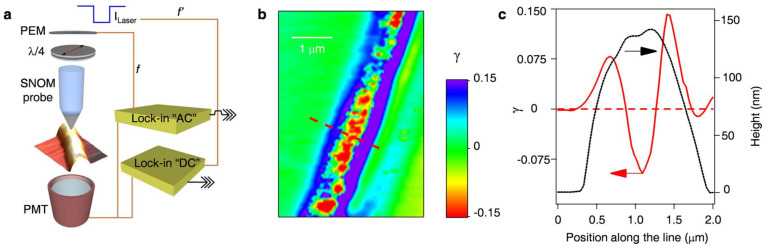
Spatial distribution of molecular orientation within electrospun polymer fibers (**a**). Nanoscale mapping of dichroic ratio reveals heterogeneous molecular alignment across the fiber cross-section, with axially oriented chains in the core and radially oriented chains near the surface (**b**). This spatial variation reflects strong elongational stretching followed by incomplete relaxation prior to solidification (**b**,**c**). (**c**) Line profile extracted along the dashed line in (**b**), showing the variation of dichroic ratio (γ) and fiber height across the fiber cross-section. The arrows schematically indicate the different preferred molecular orientations across the fiber cross-section, consistent with the sign change of the dichroic ratio (**c**). Adapted with permission from Andrea Camposeo [147], Macromolecules https://pubs.acs.org/doi/10.1021/ma500390v (Accessed on 4 March 2026). Copyright © American Chemical Society.

The competition between deformation induced chain stretching and chemical structure-governed relaxation determines under highly nonequilibrium conditions of electrospinning whether molecular orientation is preserved or disappeared before solidification [85,145]. When the characteristic relaxation time of the polymer exceeds the effective deformation time of the jet, entanglements and intermolecular associations act as transient elastic junctions trapping kinetically stretched chain conformations [82,123,146]. Direct experimental mapping of molecular orientation frozen during electrospinning is shown in Figure 3. This mechanism explains the frequent observation of orientation induced stiffness, residual stress, and changed thermal transitions in electrospun fibers.

The chain relaxation is not just a generic timing issue; it is rooted in the chemical structure of the macromolecule. The internal stiffness of the backbone, the bulkiness of the side group, and the strength of the hydrogen bonds all determine how much of molecular memory the final fiber will have [10,77,85]. This explains why electropsun mats often show higher stiffness and different melting temperatures than traditional films. The understanding of how molecules become trapped leads directly to our next Section 2.5.

### 2.5. Polymer–Solvent Interactions and Segmental Mobility

Polymer–solvent interactions provide a chemically defined pathway through which molecular mobility, relaxation behavior, and solidification dynamics become coupled during electrospinning [85,90,108,144]. Unlike conventional polymer processing, electrospinning involves continuous solvent loss along a rapidly stretching jet, so the local solvent environment evolves on a timescale comparable to deformation itself. As a result, polymer chains move from a highly solvated and mobile state toward a progressively concentrated state in which segmental motion becomes increasingly restricted. The rate and manner of this mobility loss strongly influence whether the jet remains stable long enough to form continuous fibers, how much of the accumulated elastic stress can relax before solidification, and which structural features are ultimately preserved in the final fiber [34,81].

#### 2.5.1. Thermodynamic Quality and Chain Expansion

The rate and uniformity of mobility loss depend strongly on polymer–solvent affinity and on the chemical nature of the polymer backbone [148,149,150]. Good solvents promote expanded chain conformations, enhance intermolecular overlap at a given concentration, and delay vitrification by maintaining higher segmental mobility during jet stretching [37,150]. Under such conditions, part of the elastic stress accumulated during elongation can relax before solidification, which often favors smoother and more uniform fibers. By contrast, poor-solvent conditions tend to promote coil contraction, faster local concentration buildup, and earlier mobility loss. This can freeze nonequilibrium chain conformations and stress distributions into the solidified fibers, thereby increasing the likelihood of structural heterogeneity, roughness, or bead formation [37,151,152].

These differences help explain why identical nominal electrospinning conditions can produce markedly different outcomes depending only on solvent choice. For example, when PAN is dissolved in high-affinity solvents such as DMF [153,154,155], the chains remain highly expanded, which generally supports stable jets and uniform fibers. If a less compatible co-solvent is introduced, polymer chains tend to contract, which reduces intermolecular overlap. As a result, even at the same overall polymer concentration, the system may no longer sustain a stable entangled network, leading to beaded or unstable fiber morphologies. Solvent quality should therefore be viewed not merely as a solubility requirement but as a molecular design variable that defines the initial conformation and connectivity of the polymer network entering the electrospinning jet.

#### 2.5.2. Segmental Mobility and the Vitrification Pathway

Once the jet has formed, the central solvent-related question becomes not only how fast solvent is removed but how the timescale of solvent loss compares with the timescales of segmental rearrangement, stress relaxation, and possible crystallization or demixing. As the polymer concentration rises, the rotational and translational freedom of local chain segments becomes progressively restricted [31,70,75,152,156]. This loss of segmental mobility marks the transition from a structurally adaptive jet to one that is being kinetically frozen. The point at which this transition occurs strongly affects how much of the deformation-induced molecular organization is retained in the final fibers. If solvent removal is sufficiently gradual, the jet may remain mobile over a significant portion of its thinning history. In that regime, accumulated elastic stress can partially relax, local packing can become more uniform, and orientation or concentration gradients can redistribute before final solidification. This tends to favor dense and structurally homogeneous fibers, even if the jet initially experiences strong stretching. High-boiling or strongly interacting solvents can support this pathway because they prolong the plasticized state of the polymer and delay mobility arrest [75,138,141,144].

A different regime emerges when solvent loss is so rapid that concentration rises faster than the chains can respond structurally. In that case, viscosity increases abruptly, segmental motion becomes arrested early, and the outer region of the jet may solidify before the interior has equilibrated [69,157,158]. This produces a vitrification-like trapping pathway in which the final fiber preserves a nonequilibrium internal state rather than a relaxed one. The resulting structure may retain frozen orientation gradients, residual stress, local density inhomogeneity, or skin–core asymmetry. The importance of volatility therefore lies not merely in accelerating drying but in shifting the balance between relaxation and fixation toward earlier kinetic arrest. This interpretation also clarifies why mobility arrest should not be described as a single event. It may occur gradually throughout the jet or non-uniformly across the jet radius, depending on solvent diffusion, external humidity, and local evaporative cooling [95,156,158]. The relevant design variable is thus not simply solvent boiling point, but the relative ordering of four timescales: solvent evaporation, segmental relaxation, phase separation, and solidification [104,105,106]. Electrospinning outcomes become more predictable when solvent choice is discussed in terms of these competing timescales rather than in terms of volatility alone [159].

#### 2.5.3. Binary Solvent Systems and Evaporation Balance

Mixed-solvent systems are especially informative because they reveal that electrospinning is governed by solvent pathways rather than by single solvent properties [160]. In a binary or ternary system, each component may influence a different part of the process: one solvent may maintain chain expansion, another may raise conductivity, and a third may modify evaporation rate or trigger demixing [158,161]. The resulting jet therefore evolves through a changing composition trajectory rather than through uniform solvent removal [65]. As the more volatile component is depleted, the remaining liquid phase may become less compatible with the polymer, more viscous, more strongly associated, or more prone to phase separation. This illustrates that mixed solvents can decouple the molecular state of the jet from its initial solution state. A formulation that begins as a homogeneous and apparently well-solvated solution may move during flight into a regime of reduced solvent quality, local concentration fluctuations, or selective solvation of different chain segments [69,162]. In some systems, this transition is useful because it allows spinnability and morphology to be tuned independently. For example, one component can support early jet formation while another delays full relaxation or induces a controlled demixing step later in flight [69,163]. In other systems, however, the same strategy can generate unstable or poorly reproducible outcomes because a small change in composition, humidity, or evaporation rate shifts the jet into a different solidification pathway [164]. Binary solvent systems should be interpreted not as static recipes, but as dynamic routes through solvent-quality space. Their utility lies in allowing the researcher to sequence events during jet evolution: first stabilize the solution, then thin the jet, then fix or restructure the morphology. This is why mixed solvents are particularly powerful for porous-fiber fabrication, yet also particularly sensitive to small experimental variations [158,165].

#### 2.5.4. Solvent-Induced Phase Separation and Internal Nanostructure

**Figure 4:**
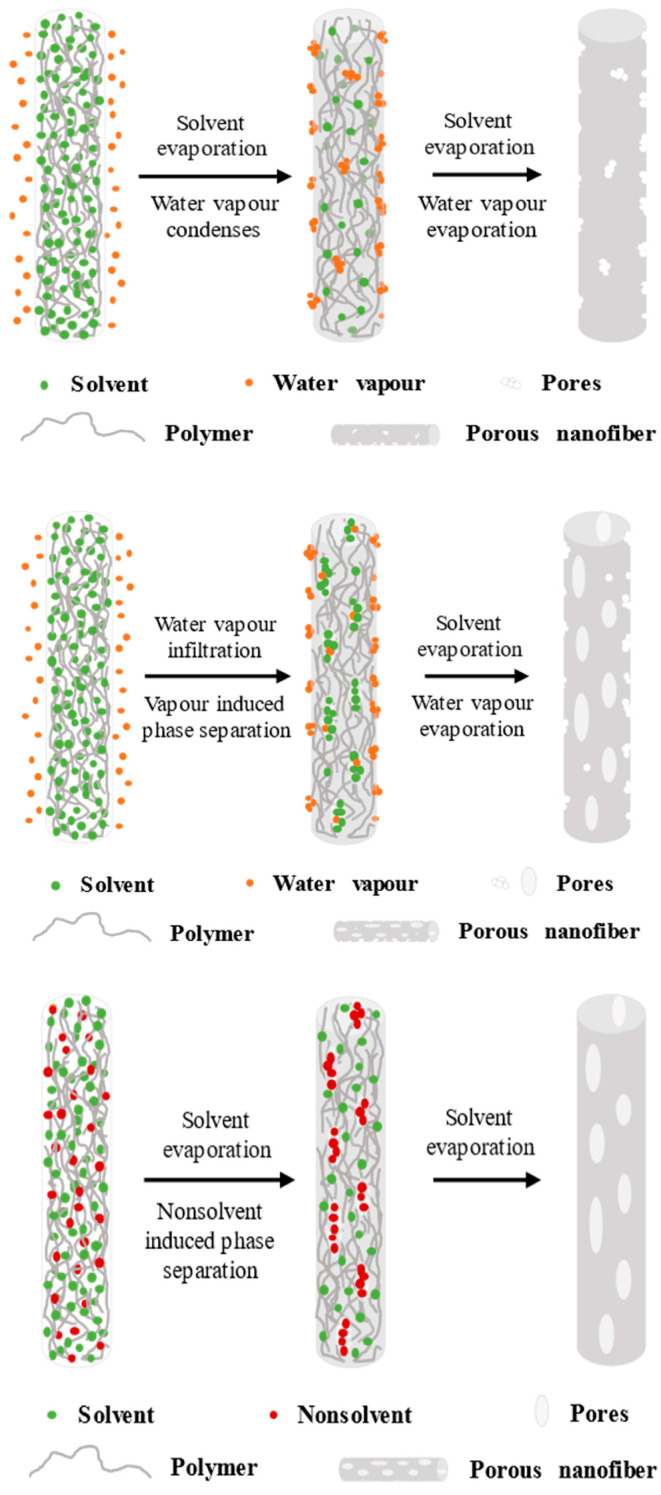
Schematic illustration of solvent-induced phase separation pathways during electrospinning. The diagram compares breath-figure (BF), vapor-induced phase separation (VIPS), and nonsolvent-induced phase separation (NIPS) mechanisms. The pore structures shown are not to scale and do not represent actual pore size, connectivity, or mechanical integrity. Instead, they are intended to qualitatively illustrate the evolution of polymer-rich and polymer-poor domains during jet solidification. Adapted from [170] under CC BY- 4.0 license.

The deepest consequence of polymer–solvent interactions appears when jet solidification becomes coupled to phase separation. Under these conditions, the evolving jet must be understood not only as a drying filament, but as a transient multicomponent system that can cross into thermodynamic instability while it is still being stretched [36,59,138,139,144,151,166]. This may occur because solvent composition changes during evaporation, because water vapor from the atmosphere acts as a non-solvent, or because diffusional imbalance creates local enrichment of a less compatible component. Once such instability develops, the jet may separate into polymer-rich and polymer-poor domains. The subsequent evolution of these domains depends on how quickly solidification arrests the structure [69,125,126]. This phase-separation perspective makes it necessary to distinguish several morphology classes that are often grouped too loosely. Smooth dense fibers arise when evaporation and relaxation remain balanced enough to avoid substantial demixing or surface freezing. Rough-surfaced fibers, by contrast, may form when the outer layer solidifies early and preserves surface corrugation, but without generating stable pore domains [167]. Surface-porous fibers require the formation and retention of polymer-poor regions near the jet surface, often through vapor-induced or breath-figure-like mechanisms. Internally porous fibers require a stronger bulk demixing pathway, such as non-solvent induced phase separation or composition-driven liquid–liquid separation inside the jet. These outcomes are related, but they are not interchangeable and should not be described as a single continuum from smooth to porous [167,168]. These mechanisms and resulting structures have also been widely discussed in the context of electrospun porous fibers formed via solvent–nonsolvent interactions and controlled phase separation [169]. Figure 4 [170] illustrates how different solvent evaporation rate and phase separation mechanisms (VIPS/NIPS/BFs) produce distinct porous structures during electrospinning. This links polymer–solvent interactions to segmental mobility and fiber solidification. These considerations illustrate that electrospinning solidification is not only a physical drying process but a chemically controlled way in which polymer–solvent interactions dictate segmental mobility, relaxation opportunities, and the preservation of nonequilibrium structures. It should be noted that the pore structures shown in Figure 4 are schematic and not intended to represent actual pore size or mechanical integrity, but rather to illustrate qualitative phase-separation pathways.

## 3. Molecular Informed Design Strategies for Electrospinning

**Figure 5:**
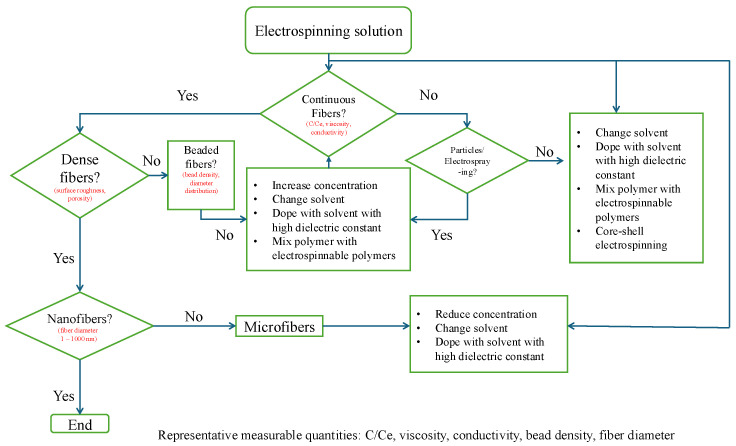
Design logic flowchart for electrospinning. The scheme outlines a practical pathway from solution state to morphology outcome, including electrospraying, beaded fibers, continuous fibers, dense fibers, and final fiber diameter. The measurable quantities shown, such as $C/C_{e}$, viscosity, conductivity, bead density, porosity, and fiber diameter, are representative experimental indicators rather than universal thresholds.

This section focuses on practical instruction instead of mechanism description and we try to translate the molecular-level contents into a clear logic for electrospinning. The following discussion emphasizes the order in which molecular and processing variables should be considered to achieve stable electrospinning outcomes. To clarify how the molecular principles discussed in Section 2 translate into practical electrospinning control, a schematic overview of the design logic adopted in this section is provided in Figure 5.

### 3.1. Molecular Design

Molecular design constitutes the primary control level in electrospinning and defines the window for the manipulation of operational parameters. The transition from random electrospraying to stable fiber formation is determined by the macromolecular architecture, especially the degree of chain entanglement and viscoelasticity according to Section 2. Therefore, polymer systems without appropriate relaxation dynamics or entanglement density cannot be electrospun through external variables adjustments [132,146,159]. This suggests that the optimization of the electrospinning process should prioritize polymer selection and solution formation over tuning of parameters from the practical perspective. Some studies have demonstrated that molecular characteristics such as molecular weight distribution and chain topology determine the boundaries of jet stability and structure formation [138,139,150,171]. Processing parameters like applied voltage or feed rate act only as secondary modulators within these molecularly defined limits. The attempts to compensate for unfavorable molecular properties by accelerating the electric field usually lead to jet instability and structural defects rather than performance enhancement. Successful fiber engineering may need to begin first with a material to ensure that the molecular foundation is compatible with the fluid dynamics of the electrospinning process [39,45,53].

### 3.2. Molecular Weight and Molecular Weight Distribution

**Figure 6:**
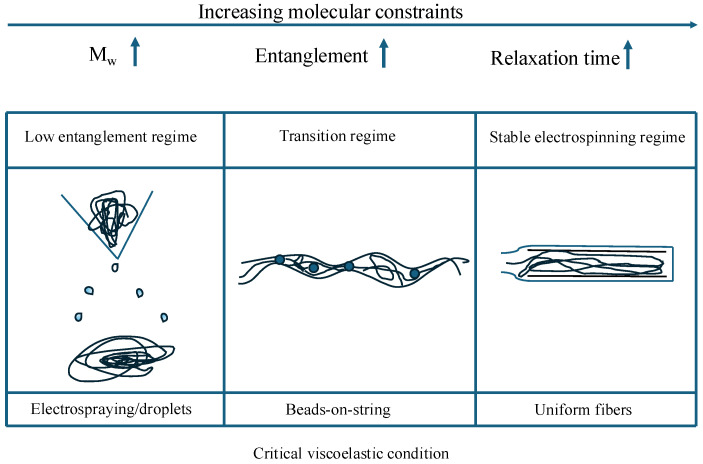
Molecularly governed stability regimes in electrospinning.The arrows indicate the progressive increase in molecular weight ($M_{w}$), chain entanglement, and relaxation time from left to right, corresponding to increasing molecular constraints and the transition from electrospraying/droplets to beads-on-string and finally to uniform fibers.

The transition from a liquid droplet to a solid fiber is perhaps the most important part of electrospinning and is usually governed by molecular weight and molecular weight distribution because of their influence on chain entanglement (see Section 2.1 and Section 2.2). For polymers such as PEO [93], PAN [172], and PCL [69], solutions prepared below this connectivity threshold typically exhibit electrospraying or bead-on-string morphologies because short chains or insufficient overlap cannot generate adequate elastic resistance. As molecular weight or concentration increases beyond the entanglement regime, the formation of a percolated viscoelastic network suppresses Rayleigh instability and enables stable jet thinning. Thus, the transition from droplets to continuous fibers reflects a rheologically defined threshold rather than a purely concentration-based boundary. The jet often fragments into droplets when using low molecular weight batches, because the short chains cannot provide enough internal friction to hold the fluid together leading to a phenomenon known as electrospraying [41]. These droplets begin to connect as the molecular weight increases forming a bead-on-string structures until a sufficiently high molecular weight finally yields smooth and uniform fibers [25,107]. The progressive transition from jet breakup to stable fiber formation as a function of molecular weight characteristics is schematically illustrated in Figure 6. Such transitions highlight that spinnability is not a binary property, but a molecularly defined window that can be expanded through appropriate control of molecular weight distribution.

However, molecular weight alone does not fully determine electrospinning behavior. Two polymer samples with similar average molecular weights may still respond quite differently under the high elongational strain rates of an electrospinning jet, which highlights the importance of molecular weight distribution [24,25,107]. A distribution containing a sufficiently long high-molecular-weight tail can contribute disproportionately to elastic stress development during jet thinning. In some polymer systems, utilizing a broader molecular weight distribution, or blending a small fraction of high-molecular-weight polymer into a lower-molecular-weight matrix, can improve spinnability and widen the practical operating window. This effect is system dependent and should not be generalized to all polymers, since its benefit depends on polymer chemistry, solvent quality, and concentration regime [23,85].

### 3.3. Molecular Architecture and Stress Transmission

Once basic spinnability is established, molecular architecture becomes a second level design variable that determines how efficiently a polymer system converts molecular connectivity into extensional stress during jet thinning [173,174]. Its role is not limited to a geometric description of chain shape. Rather, architecture controls how chains overlap in solution, how readily an entanglement supporting network develops, and how stress is stored or dissipated under the extreme deformation rates of electrospinning [34,123]. For this reason, architecture influences not only whether continuous fibers can be formed but also how broad or narrow the accessible processing window remains once spinning begins.

Linear polymers generally provide the most efficient intermolecular overlap per unit molecular weight because their more extended conformations favor the formation of a connected transient network. But branched and star-shaped architectures often occupy a smaller hydrodynamic volume at comparable molecular weight and can therefore require higher concentrations to reach an equivalent degree of intermolecular connectivity [110,131,136]. The practical consequence is that two systems with similar average molecular weight may still exhibit very different electrospinning behavior if one is linear and the other is topologically compact. The relevant comparison is not simply molecular weight itself, but the relationship among topology, overlap efficiency, and the concentration required to sustain a deformation-resistant network [175].

However, the effect of branching should not be reduced to a simple loss of entanglement efficiency. Branched architectures also modify how the solution relaxes [129,176]. In addition to the relaxation modes present in linear chains, branched and star-shaped macromolecules may exhibit topology-dependent processes such as arm retraction, branch-point fluctuations, and constrained segmental rearrangement [130,177]. These additional modes become important because electrospinning subjects the jet to a deformation history that is both rapid and highly nonequilibrium. If the dominant architectural relaxation modes are slow compared with the deformation and solidification timescale, the system may retain elastic stress long enough to suppress capillary breakup and support stable fiber formation. If they are fast, the accumulated stress may dissipate before the jet becomes structurally fixed, even when apparent entanglement is present. Hyperbranched and dendritic polymers represent the limiting case particularly well [119,120]: despite their potentially high nominal molecular weights, their globular topology often makes them poor standalone electrospinning candidates unless they are blended with linear high-molecular-weight chains or reinforced through strong associative interactions.

Block copolymers [1,103,124] add a further level of complexity because their architecture couples electrospinnability to internal compositional organization. The connectivity between chemically distinct blocks links jet stretching to the possibility of microphase separation, interdomain alignment, and kinetically trapped internal morphology. During electrospinning, solvent evaporation and elongational deformation occur on timescales comparable to or faster than those required for full structural equilibration [61]. As a result, the final nanostructure of electrospun block copolymer fibers is often not an equilibrium morphology, but a frozen record of competing processes including chain stretching, selective solvation, interblock incompatibility, and solidification rate. Parameters such as block ratio, segregation strength, and solvent selectivity therefore influence not only whether the jet remains stable but also whether the resulting fibers display internal nanodomains, anisotropic phase orientation, compositional asymmetry, or hierarchical texture [178,179].

### 3.4. Solvent Effects on Relaxation and Solidification

Solvent selection plays a central role in determining whether molecular orientation and internal structures generated during electrospinning are contained or relaxed prior to solidification [148,151]. As discussed in Section 2, polymer–solvent interactions influence segmental mobility along the jet trajectory and then affect the balance between molecular relaxation and structure fixation during electrospinning process. Polymer solutions prepared in high affinity solvents tend to remain plasticized for a longer time during the jet’s flight. This extended mobility allows for a partial relaxation of elastic stresses and molecular rearrangement commonly associated with smooth and relatively dense fiber morphologies [123,152]. Solvents with high boiling points and strong affinity for the polymer act as plasticizers, maintaining segmental mobility for a longer duration during electrospinning. In contrast, solvents with lower polymer affinity (poor solvents) promote a rapid increase in solution viscosity and accelerate mobility loss, leading to earlier solidification and the preservation of nonequilibrium conformations. These conditions will promote highly extended chain states or internally heterogeneous structure within electrospun fibers [140]. Mixed solvent systems provide for an additional point by allowing evaporation behavior and solubility to be adjusted independently. The combination of a volatile solvent with poorer solvent with higher boiling point can induce phase separation during jet elongation. This is a strategy for production of porous fiber morphologies. Varying the ratio of volatile solvents like tetrahydrofuran to less volatile solvent like dimethylformamide for polymer systems such as PS [151] or PMMA [58] allows for the systematic tuning of fiber morphology from smooth to highly porous. The relative rates of solvent loss and polymer relaxation ultimately determine whether transient molecular arrangements are relaxed or kinetically trapped. Solvent choice variation can be a practical means of fixing specific molecular and structural features that arise during jet elongation accordingly.

### 3.5. Processing Parameters

Processing parameters [44] should not be interpreted as primary determinants of electrospinnability, but as operational variables that act within material defined limits [180]. Once molecular weight distribution, chain architecture, solvent quality, and intermolecular connectivity establish a jet that is at least potentially spinnable, parameters such as applied voltage, feed rate, and tip-to-collector distance determine how that jet actually traverses the pathway from fluid emission to structural fixation [104,150]. Their importance therefore lies less in creating stability from an intrinsically unsuitable system than in redistributing the relative influence of stretching, relaxation, solvent loss, and deposition over the available flight time. Processing conditions influence electrospinning primarily by altering the ordering and intensity of competing events. Applied voltage alters the electrical stress imposed on the jet and therefore the rate of elongational deformation. Feed rate controls how much material must be stretched and dried per unit time. Tip-to-collector distance determines the effective residence time available for thinning, charge redistribution, evaporation, and solidification. None of these parameters acts independently of the molecular state of the solution [39,40,45]. Instead, each one shifts the balance between stress generation and stress dissipation in a way that is only meaningful relative to the entanglement density, relaxation spectrum, and solvent-controlled mobility window of the specific polymer system. Typical electrospinning conditions are often reported in the ranges of applied voltage (~10–25 kV), feed rate (~0.1–1 mL h^−1^), and tip-to-collector distance (~10–20 cm). However, these values are not universal and must be interpreted relative to the molecular and solution properties of the system, including entanglement density, viscosity, conductivity, and solvent evaporation rate [11,36,37,39].

Applied voltage is often discussed as a direct route to finer fibers, but its effect is more conditional than this common interpretation suggests [33]. In a well-entangled and sufficiently conductive system, increasing voltage can indeed intensify electrostatic stretching, reduce fiber diameter, and improve jet initiation. However, the same increase in electrical forcing can also amplify bending instability, whipping amplitude, or local stress concentration when the solution is only marginally connected. In such cases, stronger forcing does not refine the jet in a controlled way; rather, it accelerates the manifestation of pre-existing molecular weakness [159]. Voltage is therefore best understood as a stress amplification parameter whose usefulness depends on whether the polymer network can store and transmit the imposed deformation without breaking into beads or droplets.

Feed rate is equally important because it governs the competition between material supply and structural maturation of the jet. A higher feed rate increases throughput, but it also illustrates that a larger volume of liquid must undergo stretching, concentration buildup, and solidification over essentially the same travel distance [181]. If the rate of solvent removal and viscoelastic stabilization cannot keep pace, the jet reaches the collector in an incompletely fixed state. The resulting consequences can include fused fibers, flattened cross-sections, residual solvent retention, or broader diameter distributions. In systems near the spinnability threshold, excessive feed rate can also suppress the apparent benefit of otherwise favorable molecular design, because the material is delivered faster than the evolving network can convert concentration increase into mechanically useful elasticity. Conversely, very low feed rates may stabilize difficult systems, but often at the cost of productivity and with increased sensitivity to tip drying or intermittent jet formation [11,36].

Tip-to-collector distance is sometimes treated as a simple geometric parameter, yet its physical role is to define the time window over which thinning, evaporation, relaxation, and fixation can occur before deposition [181]. A longer distance can be beneficial if the jet requires additional time to concentrate and solidify, particularly in systems with slower solvent loss or longer-lived segmental mobility. But this does not mean that increasing distance is intrinsically favorable. If molecular relaxation is too rapid, the additional flight time may simply allow more stress dissipation without preserving extra orientation. If evaporation is already extremely fast, the jet may become kinetically trapped well before reaching the collector, in which case extra distance does little to improve morphology [182]. Distance therefore interacts strongly with solvent volatility and relaxation time, and its effect should be interpreted as a temporal matching variable rather than as a stand-alone optimization parameter [34,123].

The apparent inconsistency of electrospinning recipes is therefore not merely an issue of experimental variability; it reflects the fact that processing parameters only acquire meaning within a specific molecular and formulation context [36,181]. What appears to be a successful operating window in one system may be inaccessible in another because the relevant balance among deformation, relaxation, and solidification has shifted. Parameter optimization should be regarded as the final stage of electrospinning design rather than the starting point. Once the solution is molecularly and thermodynamically tuned to support continuous jet formation, processing parameters can be used to fine-tune fiber diameter, deposition behavior, productivity, and morphology uniformity. When that foundation is absent, however, adjusting external settings rarely compensates for insufficient entanglement, poorly matched solvent quality, or unfavorable relaxation dynamics [182,183]. In this sense, processing parameters do not replace molecular design; they operationalize it by selecting how a material defined electrospinning window is traversed in practice. For clarity, the respective roles of molecular, solvent, and processing variables discussed in this section are summarized in Table 2.

## 4. Discussion and Outlook

### 4.1. Reproducibility Challenges in Electrospinning

Electrospinning is frequently described as a technique with limited reproducibility, especially when nominally similar formulations produce different jet behavior or fiber morphologies across different laboratories [36,37]. This inconsistency is often explained in operational terms, such as variation in voltage, flow rate, humidity, or collector setup. Although such factors undoubtedly influence the final outcome, a parameter-centered explanation remains incomplete because it treats variability mainly as a problem of process control rather than as a consequence of how close the material system operates to its intrinsic stability limits [36,104,139,140,184]. Reproducibility is fundamentally a question of whether the same solution state is being reproduced before spinning begins. A polymer formulation is not fully specified by concentration and solvent identity alone. Its effective electrospinning behavior also depends on molecular weight distribution, chain architecture, degree of degradation, dissolution history, aging time, residual aggregation, and the strength of polymer–solvent and polymer–polymer interactions. Small differences in any of these features can shift the balance among overlap, entanglement, relaxation, and solidification even when nominal recipe parameters remain unchanged. As a result, two solutions that appear compositionally identical may in fact enter the electric field with different capacities for stress transmission and jet stabilization [22,49,74,82].

This sensitivity explains why reported “working windows” are often broad yet difficult to transfer. In many cases, the apparent success of a parameter set is conditional on an unreported molecular state that is not reproduced elsewhere. What appears to be disagreement about optimal voltage, concentration, or flow rate is therefore often disagreement about where the underlying solution lies relative to its entanglement threshold, relaxation spectrum, and solvent-controlled mobility window [123]. Once these molecular constraints are recognized, the literature becomes more interpretable: the instability is not random but reflects that the system is being driven near a boundary separating droplets, beads, and continuous fibers [74,180]. A similar argument applies to scale-up. Scaling electrospinning is not only a matter of increasing throughput or redesigning collectors; it also requires maintaining the same molecularly relevant balance among connectivity, charge response, evaporation, and fixation under altered processing conditions. If these balances change, the scaled process may no longer traverse the same physical pathway as the laboratory-scale jet, even when the hardware appears analogous. Reproducibility and scalability are therefore linked by a common requirement: the molecular state of the material must be defined and preserved with sufficient rigor for the process window to remain physically comparable [185,186]. This suggests that electrospinning reproducibility should be reframed less as a problem of empirical parameter inconsistency and more as a problem of incomplete material-state specification. More systematic reporting of molecular descriptors, solution history, and solvent-mediated structural evolution would narrow the interpretive gap between studies and make process transfer more rational.

### 4.2. Scalability and Process Robustness

The difficulty of scaling electrospinning beyond laboratory conditions due to increased instability or reduced uniformity at higher production rates is widely acknowledged [187,188,189]. Scale-up challenges are often framed in terms of equipment limitations, such as jet–jet interference or electric field distortion. While these factors undoubtedly contribute to process complexity, they do not fully explain why nominally similar hardware configurations can yield markedly different outcomes across polymer systems. Many publications [36,37,82,111] have shown that polymer formulations exhibiting stable electrospinning at small scales may become highly sensitive to minor fluctuations when operated under high-rate conditions. Small variations like flow rate, electric field strength, or ambient conditions can lead to abrupt transitions from continuous fiber formation to bead formation or electrospraying [84,106]. This behavior suggests that many electrospinning systems operate close to molecularly imposed stability boundaries, where robustness is limited by entanglement density and relaxation dynamics rather than by hardware design. The polymer systems reported to exhibit broader molecular weight distributions, more gradual solvent evaporation profiles, or slower relaxation dynamics often display enhanced tolerance to processing perturbations. In these cases, stable jet behavior is maintained over a wider range of operating conditions, enabling more reliable scale-up [123]. These observations imply that scalability in electrospinning is closely linked to molecular design choices that expand the accessible processing window, rather than to equipment-based solutions alone.

### 4.3. Applications for Validation of Molecular Design Principles

Although electrospinning research is often motivated by application-specific performance targets, comparisons across different application domains reveal common molecular dependencies that extend beyond individual end uses. In filtration and separation applications, fiber uniformity and pore structure are repeatedly correlated with polymer molecular weight, entanglement density, and solvent-mediated solidification behavior [190,191]. Similar trends are observed across different polymer systems, despite variations in device configuration or testing protocols. Energy-related applications provide further illustrative examples. Electrospun polymer nanofibers, particularly those based on PAN [190], are widely employed as precursors for production of carbon nanofibers. Numerous studies [74,140,192] report that molecular weight distribution and chain architecture influence not only electrospinning stability but also stabilization behavior and structural evolution during subsequent thermal treatments. These outcomes highlight that molecular characteristics established during electrospinning can propagate through downstream processing steps. These application-specific constraints are summarized in Table 3, highlighting how different end uses impose distinct tolerance requirements on electrospinning outcomes rather than defining performance targets.

Biomedical fibrous scaffolds offer a contrasting yet complementary case. While performance metrics often emphasize biological response, reported differences in fiber morphology, mechanical compliance, and degradation behavior are frequently associated with polymer chemistry, molecular architecture, and solvent choice [191,193,194]. Across these application areas, successful electrospinning outcomes consistently reflect molecularly governed structure formation rather than empirical parameter optimization, reinforcing the broader relevance of a macromolecular design perspective.

### 4.4. Methodological Boundaries and Open Challenges

Although the macromolecular design framework developed in this review provides a useful basis for interpreting many electrospinning phenomena, its explanatory power has clear boundaries. Its main strength lies in identifying the material-side constraints that determine whether a polymer solution can generate a connected, deformation-resistant jet and how that jet is likely to relax or solidify [90,123,184]. It is therefore most effective for framing electrospinning as a problem of molecular connectivity, relaxation, and solvent-mediated fixation. However, this perspective is not intended to replace detailed electrohydrodynamic descriptions of charged jets, nor does it by itself resolve local electric field heterogeneity, charge transport, space-charge effects, or full multiphysics coupling under extreme processing conditions. A second boundary is that not all electrospinnable systems are stabilized through the same molecular mechanisms. The present framework is most directly applicable to polymer solutions in which entanglement, transient association, or topology-dependent relaxation provide the dominant basis for jet stability. Systems based on low-molecular-weight species, reactive oligomers, highly supramolecular assemblies, or particle-loaded fluids may follow different stabilization mechanisms. In such cases, electrospinning may transition toward electrospraying or produce beads or particles rather than continuous fibers, unless additional interactions (e.g., rapid curing, strong associations, or particle networks) provide sufficient cohesion. Electrospinning may depend less on classical chain connectivity and more on reaction-induced solidification, interparticle interactions, field-responsive aggregation, or other nonclassical stabilization routes [195,196].

These distinctions are important because they define where molecular design can be used predictively and where it remains mainly interpretive. A framework that is too broad risks becoming descriptive rather than mechanistic, whereas one that is too narrow cannot account for the diversity of electrospinnable formulations now being reported. One of the central open challenges is therefore to determine which molecular descriptors are broadly transferable across chemically distinct systems and which remain specific to a given polymer family, solvent class, or stabilization mechanism. A related challenge is the lack of a sufficiently integrated language connecting macromolecular descriptors to experimentally measurable solution-state quantities and then to jet outcomes. Parameters such as overlap concentration, entanglement concentration, relaxation time, conductivity, and solvent-quality-dependent conformation are often discussed separately rather than combined into a predictive framework [75,197]. As a result, electrospinning research still relies heavily on retrospective interpretation rather than prospective design. Future progress will likely depend on tighter integration of polymer physics, extensional rheology, solvent thermodynamics, and electrohydrodynamic modeling, so that molecular design principles can be translated into more quantitative and transferable criteria for jet stability and morphology selection [23,27,174]. The main limitation of the present framework is also its main opportunity. It does not yet constitute a universal predictive theory of electrospinning, but it helps define the problem more clearly: which aspects of electrospinning variability arise from molecular state, which arise from field-driven process dynamics, and where the two become inseparable. Clarifying this boundary is essential if the field is to move from plausible post hoc explanation toward genuinely predictive nanofiber design [186].

### 4.5. Outlook

The broader implication of this review is that electrospinning is likely to advance most effectively when it is approached less as an empirically optimized technique and more as a molecularly constrained route to nonequilibrium fiber formation. Such a shift does not diminish the importance of processing innovation; rather, it places processing within a clearer physical framework in which polymer architecture, connectivity, relaxation, and solvent-mediated structural evolution define the feasible design space before operational refinement begins [6,21,36,37]. Several directions appear particularly important for future progress. First, the field would benefit from more systematic use of experimentally accessible molecular descriptors that can bridge polymer physics and electrospinning practice. Parameters such as overlap concentration, entanglement concentration, intrinsic-viscosity-based overlap metrics, relaxation time, and solvent-quality-sensitive conformation measures are more informative than nominal concentration or average molecular weight alone when comparing systems across studies. A future electrospinning literature built around such descriptors would be better positioned to generate transferable design rules rather than system-specific recipes [198,199].

Second, morphology development should increasingly be treated as a coupled relaxation–evaporation–phase-separation problem rather than as a simple consequence of drying rate or electric stretching. This is particularly important for porous fibers, multicomponent systems, and block copolymer formulations, where internal structure is often determined by the ordering of demixing, diffusion, and vitrification events [186]. Greater integration of these concepts would help connect external morphology, internal nanostructure, and material performance in a more predictive way [200].

Third, the next stage of electrospinning research should move toward design maps that link molecular descriptors to accessible jet regimes and morphology classes. Such maps would not eliminate system specificity, but they could reduce reliance on purely empirical optimization by clarifying which changes in formulation are likely to shift a system from electrospraying to bead formation, from bead formation to stable fibers, or from dense fibers to porous and internally structured morphologies [186]. The same framework could also improve the rational design of scale-up strategies by identifying which material-side constraints must remain invariant when throughput and geometry are altered. The long-term value of a macromolecular design perspective is not only explanatory but organizational. It helps reframe electrospinning from a broad collection of successful case studies into a more coherent field with shared descriptors, clearer boundaries, and more realistic expectations of transferability. If this transition is achieved, electrospinning may increasingly serve not only as a versatile fabrication route but also as a platform for testing how polymers respond to coupled deformation, solvent loss, and structural arrest under strongly nonequilibrium conditions [200,201].

## 5. Conclusions

Electrospinning is influenced not only by externally adjustable processing parameters but, more fundamentally, by macromolecular features that determine whether a polymer solution can sustain elongational deformation, resist capillary breakup, and solidify into continuous fibers. Chain entanglement, molecular weight distribution, molecular architecture, relaxation behavior, and polymer–solvent interactions are the primary molecular descriptors controlling these transitions. Stable fiber formation cannot be predicted solely from average molecular weight or nominal concentration but depends on whether the solution develops an entanglement-supporting and deformation-resistant network under the specific solvent conditions used for spinning. Molecular architecture further affects electrospinning by altering both overlap efficiency and relaxation behavior, while solvent-controlled solidification determines whether transient molecular organization is relaxed or kinetically trapped in the final fiber. Fiber morphology should be regarded as the outcome of coupled competition among deformation, relaxation, evaporation, diffusion, phase separation, and solidification, rather than as a simple result of electric stretching or drying rate. This perspective is particularly important for interpreting porous, rough, and internally structured fibers.

Challenges often described as issues of reproducibility or scale-up are more accurately attributed to incomplete specification of the material state. When the molecular state of the polymer solution is insufficiently defined, electrospinning behavior becomes inconsistent and difficult to replicate across studies. In contrast, considering molecular descriptors as primary design variables makes process optimization more understandable and robust. Adopting a clearer molecular perspective can advance electrospinning beyond empirical parameter screening toward more predictive, reproducible, and transferable nanofiber design.

## Figures and Tables

**Table 1:** Molecular design parameters governing electrospinning behavior.

Molecular Parameter	Molecular-Level Effect	Impact on Electrospinning Behavior	Representative Polymer Systems
Molecular weight ($M_{w}$)	Increases chain overlap and entanglement	Enables stable jet formation above $C_{e}$	PEO [74,76], PAN [96,97], PCL [69,70,71,72]
Molecular weight distribution (PDI)	Broadens relaxation spectrum	Suppresses bead formation via elastic stress storage	PAN [96,97], PET [98,99]
Polymer architecture	Alters entanglement efficiency and stress transmission	Modulates jet stability and internal fiber structure	Linear vs. star PCL [69,70,71,72], block copolymers [102,103]
Intermolecular interactions	Restricts segmental mobility	Extends relaxation time and stabilizes jet	Poly(vinyl alcohol) (PVA) [104,105,106], chitosan [92,93]
Polymer–solvent affinity	Controls coil expansion and vitrification	Determines relaxation vs. structure freezing	PCL/chloroform vs. PCL/DMF [69,70,71,72]

**Table 2:** The respective roles of molecular, solvent, and processing variables in electrospinning.

Control Level	Key Variables	Primary Role in Electrospinning
Molecular weight and distribution	$M_{w}$, MWD	Define spinnability window
Molecular architecture	Chain topology, block structure	Control stress transmission and internal structure
Solvent environment	Solvent quality, volatility	Govern relaxation and solidification
Processing parameters	Voltage, flow rate, distance	Fine-tune fiber dimensions and deposition

**Table 3:** Representative application domains illustrating how application requirements impose boundary conditions on electrospinning rather than serving as performance targets.

Application Domain	Key Tolerance Requirement	Consequence for Electrospinning
Filtration membranes	Narrow fiber diameter distribution	Requires stable jet and low fluctuation
Carbon nanofiber precursors	Structural integrity during thermal treatment	Sensitive to MWD and chain continuity
Biomedical scaffolds	Mechanical compliance and degradation control	Sensitive to polymer chemistry and solvent history

## Data Availability

The original contributions presented in this study are included in the article. Further inquiries can be directed to the corresponding authors.
